# Computation Provides a Virtual Recording of Auditory Signaling

**DOI:** 10.1371/journal.pbio.0030026

**Published:** 2005-01-04

A small rodent rustles through a field in the still night, making just enough noise to betray its location to a circling barn owl. A female frog sits on the bank of a pond amid a cacophony of courting bullfrogs, immune to the mating calls of all but her own species. Thanks to a sophisticated sensory processing system, animals can cut through a vast array of ambient auditory stimuli to extract meaningful information that allows them to tell where a sound came from, for example, or whether they should respond to a particular mating call.

An acoustic stimulus arrives at the ear as sound energy in the form of air pressure fluctuations. The sound signal triggers oscillations in mechanical resonators such as the eardrum and hair sensilla. These oscillations convert sound energy into mechanical energy, opening ion channels in auditory receptor cells and producing electrical currents that change the neuron's membrane potential. This, in turn, produces the action potential that carries the sound signal to the brain. This multistep signal transduction process takes less than a millisecond, but exactly how it occurs at this time scale remains obscure. Direct measurements of the individual steps can't be made without destroying the mechanical structure; consequently, most measurements are taken downstream of the mechanical oscillations at locations like the auditory nerve. Likewise, the temporal resolution of most stimulus–response trials is far too imprecise to analyze processing at the sub-millisecond level.

Given these experimental limitations, Tim Gollisch and Andreas Herz turned to computational methods and showed that it's possible to reveal the individual steps of complex signal processing by analyzing the output activity alone. Using grasshopper auditory receptors as models, the authors identified the individual signal-processing steps from eardrum vibrations to electrical potential within a sub-millisecond time frame and propose a model for auditory signaling.

The crucial step in their study is the search for those sets of inputs (stimuli) that would yield a given *fixed* output (response). To get the parameters to describe the final output, the authors generated a sound stimulus (two short clicks) and recorded axon responses of receptor neurons in a grasshopper auditory nerve. From these recordings, they defined the fixed output as the probability of a receptor neuron firing a single action potential. They then asked how the various parameters, which were associated with different time scales, could produce the same predefined firing probability.[Fig pbio-0030026-g001]

**Figure pbio-0030026-g001:**
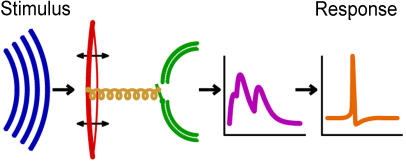
A schematic representation of auditory signaling

By varying the stimulus parameters and comparing the obtained values within their mathematical framework—and making certain assumptions, for example, that the steps signal through a “feedforward” process—they could then tease out the individual processing steps that contribute to the desired output within the required time frame. With this approach, Gollisch and Herz disentangled individual steps of two consecutive integration processes—which they conclude are the mechanical resonance of the eardrum and the electrical integration of the receptor neuron—down to the microsecond level. Surprisingly, this fine temporal resolution is achieved even though the neuron's action potentials jitter by about one millisecond.

Thus, using just the final output, this approach can extract the temporal details of the individual processes that contribute to the chain of auditory transduction events. While this method is best-suited for deconstructing unidirectional pathways, the authors suggest it could also help separate “feedforward” from feedback signaling components, especially when feedback is triggered by the final steps. But since many sensory systems share the same basic signal-processing steps, this method is likely applicable to a broad range of problems.

